# Animal Assisted Interventions in the Green Care Framework: A Literature Review

**DOI:** 10.3390/ijerph18189431

**Published:** 2021-09-07

**Authors:** Morgana Galardi, Marta De Santis, Roberta Moruzzo, Franco Mutinelli, Laura Contalbrigo

**Affiliations:** 1 National Reference Centre for Animal Assisted Interventions, Istituto Zooprofilattico Sperimentale delle Venezie, Viale dell’Università 10, 35020 Legnaro, Italy; mdesantis@izsvenezie.it (M.D.S.); fmutinelli@izsvenezie.it (F.M.); lcontalbrigo@izsvenezie.it (L.C.); 2 Department of Veterinary Science, University of Pisa, Viale delle Piagge 2, 56122 Pisa, Italy; roberta.moruzzo@unipi.it

**Keywords:** animal assisted interventions, green care, literature review, ethical approach, animal welfare, human–animal interaction

## Abstract

Green Care (GC) and Animal Assisted Interventions (AAI) are recognised practices useful to enhance the wellbeing of people through interaction with nature and animals. This study aims at understanding the interconnections between GC and AAI by analysing deeply which interaction with animals is conducted. Therefore, we carried out a literature search through Web of Science and Google Scholar that allowed retrieval of 993 records; after the PRISMA selection process, 42 were included. Relevant information was extracted: year of publication, geographical location, objectives, settings in agricultural environment, animal species, characteristics of users involved, type of human–animal interaction, coexistence of other activities without animals, animal health and welfare issues. From the review emerged that research on GC with animals is common in high-income countries and that the line between AAI and occupational therapy is often vague. Moreover, the most common setting for these interventions appears to be the farm, and frequently animals involved are not selected according to their ethological characteristics. Users in this context are extremely various and not only involved in activities with animals. Within the included studies, we noted a lack in the consideration of animal welfare that indicates the need for increased awareness among practitioners and a more ethical approach when animals are involved.

## 1. Introduction

Green Care is a complex concept identified by a simple phrase. Since the early 2000s, it has started to appear in the scientific literature and became subject of research conducted by the European Cooperation in Science and Technology network that, by means of expert consultation, defined the Green Care conceptual framework [1]. Nowadays, we use Green Care as an umbrella term to include all the approaches in which the resources of nature provide health and wellbeing to people [2].

In this paper, our main interest is to investigate a specific branch of the Green Care framework, i.e., Animal Assisted Interventions (AAI). The field of AAI is usually considered independent because its development began in the 1960s with Boris Levinson experiences [3], and the scientific literature on AAI is mostly focused on the benefits of human–animal interaction [4]. An AAI can be defined as “a goal oriented and structured intervention that intentionally includes or incorporates animals in health, education and human services for the purpose of therapeutic gains in humans” [5]. This definition was drawn up by the International Association of Human-Animal Interaction Organizations (IAHAIO), an entity that has been engaged in research and the practice of human–animal bonds and AAI from the early 1990s, including members from all over the world.

Nevertheless, when the Green Care concept started to be defined, it embedded AAI within the range of approaches in which natural resources (animals, in this case) are beneficial to human health and wellbeing [6]. From that moment, scientific literature concerning Green Care has been extremely various in the terminology spectrum used and frequently intertwined with AAI [7]. Obviously, this correspondence is not one-to-one: not all Green Care activities involve animals and their relationship with humans (e.g., horticulture), and at the same time a large extent of scientific literature on AAI does not imply a natural setting, or it does not include the effects of the natural setting within the purposes of the study.

Therefore, we conducted a literature review in order to investigate the role of the interaction with animals in the Green Care context. Our aim is to clarify the interconnection between Green Care and AAI and to understand deeply what kind of interaction with animals is conducted in the Green Care context, focusing on the users, the settings and the species involved.

## 2. Methods

### 2.1. Eligibility Criteria

We considered only records describing activities or interventions that involved both the agricultural environment and interaction with animals (to which from now on, we will refer with the term “Green Care with animals”). Given the relatively recent conceptualization of Green Care, we considered only the timespan 2000–2020. Only literature published in English was included. Review articles were excluded to ensure to rely only on primary data.

### 2.2. Databases and Search Strategy

The literature search was carried out in April 2020 through Web of Science and Google Scholar. Search strings are reported in Table 1. The total number of records resulting from the Web of Science platform (613) and the first 400 results of Google Scholar (sorted by relevance) were retrieved for the screening phase.

### 2.3. Data Collection Process

Two reviewers conducted the screening phases. The records (*n* = 993) were exported into EPPI-Reviewer Web software in order to remove duplicates and then titles and abstracts were screened to remove non-relevant ones. Subsequently, the remaining 67 records underwent full text screening. Finally, from the included records (*n* = 42) we collected in a Microsoft Excel Table the following information: year of publication and geographical location of the study, objectives of the study, settings in agricultural environment, animal species and characteristics of the users involved, kind of human–animal interaction, other activities conducted without animals, animal welfare issues and health perspectives.

## 3. Results

### 3.1. Overview

**Figure 1:**
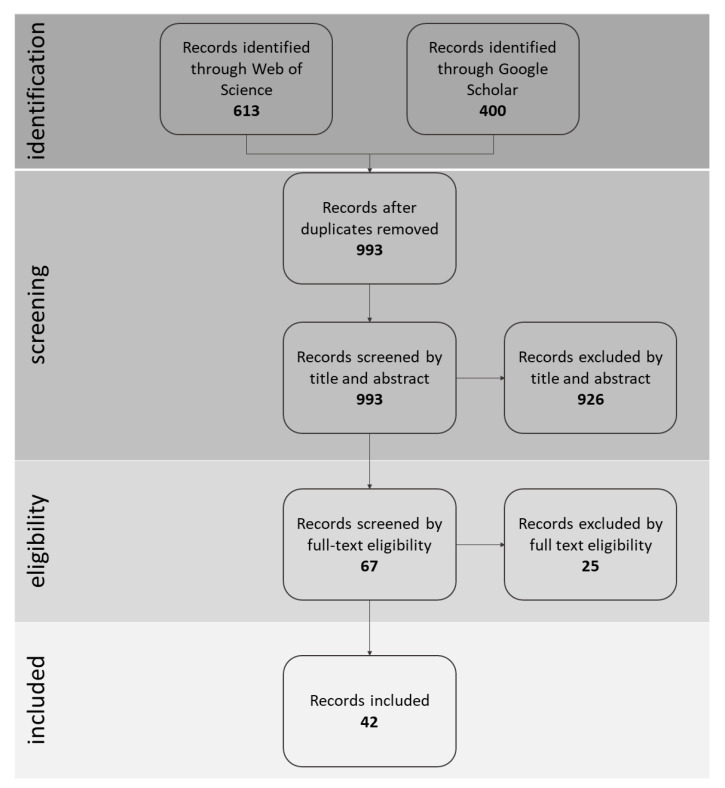
PRISMA inspired flow diagram.3.1. Year of publication and geographical distribution of the studies.

The selection process is summarized in Figure 1 with a PRISMA inspired flow diagram [8]. A total of 42 records were included in this review: 33 peer reviewed journal articles, six book chapters published by Springer, one peer reviewed conference proceeding, one book and one report. All publications are listed in the Appendix A. A synthesis of the information extracted is reported below.

**Figure 2:**
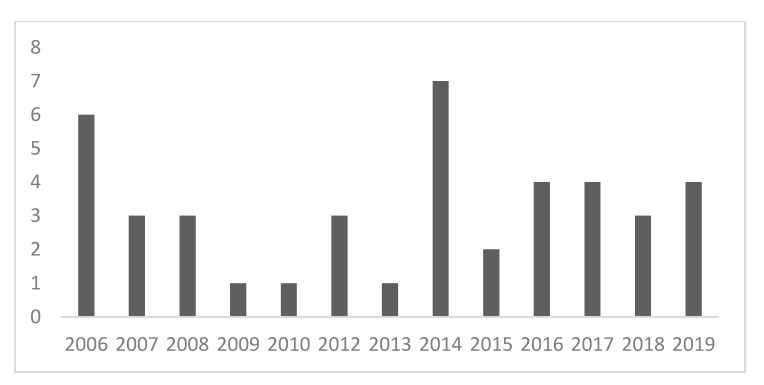
Number of records per year (*n* = 42).

Most records included in this review were published in 2006 and in 2014 (Figure 2). Of the six records considered for 2006, five were collected in a book titled “Farming for Health” [9]. The year with the maximum number of papers about human–animal interaction in the Green Care framework published by different research groups was 2014.

**Figure 3:**
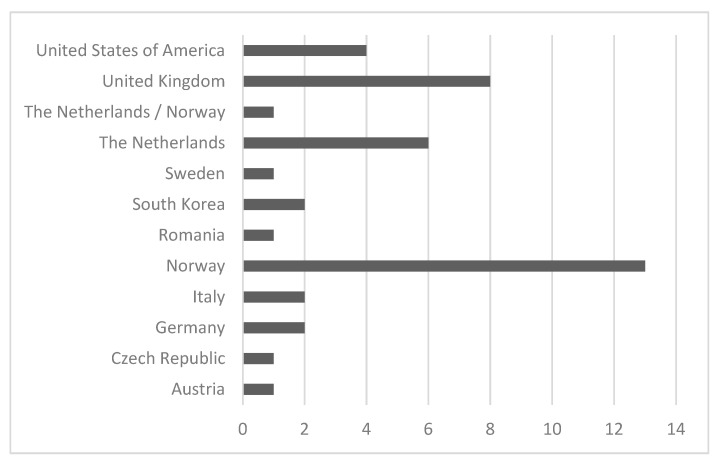
Distribution of records per country (*n* = 42).

As for geographical location, most studies were conducted in Europe, especially in Norway (30.9%), the United Kingdom (19%) and the Netherlands (14.2%). Outside of European countries, the studies fulfilling the predefined eligibility criteria were conducted in the United States of America (9.5%) and South Korea (4.8%) (Figure 3). 

Comparing years of publication and countries, we can see that some nations published on this theme only in one year (Austria, Germany and Sweden in 2006 and Italy in 2019), while others, such as Norway, the Netherlands and the United Kingdom, published more or less every year. We did not find a trend of publication related to the time in any country.

### 3.2. Objectives of the Studies

Within the records analysed, around 36% (*n* = 15) aimed at mapping Green Care providers and/or at describing Green Care practices in a geographical area (e.g., a country or a region) [10,11,12,13,14,15,16,17,18,19,20,21,22,23,24]. The remaining approximately 64% (*n* = 27) described the perceived benefits of Green Care with animals programs by both users and providers. In detail, in six of these studies, Green Care with animals programs were compared to other approaches (with the same users’ category) to highlight their benefits [25,26,27,28,29,30]. In three records, the main focus was the evaluation of the efficacy of the Green Care with animals programs, but experimental designs lack of control groups [31,32,33]. Other 13 records analysed the benefits of Green Care with animals for users by means of qualitative methods [34,35,36,37,38,39,40,41,42,43,44,45,46]. Finally, five of these records asked for the opinion of providers on the efficacy and benefits of Green Care with animals programs [47,48,49,50,51].

### 3.3. Settings in Agricultural Environment

**Figure 4:**
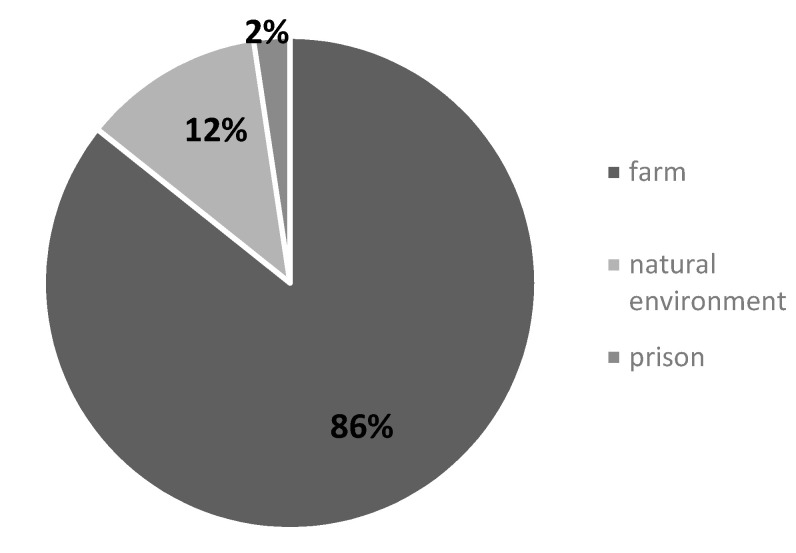
Percentage of different settings (*n* = 42).

Studies included in our review were mainly carried out in farms (Figure 4). Just a few records mentioned a “natural environment” in general [11,12,15,34,39] and only one was conducted in the prison context [19].

### 3.4. Animal Species Involved

In 25 records, the animal species involved were more than four or generally identified as “farm animals”. Other eight records did not even specify the animal species involved, even if the users interacted directly with them [17,18,20,22,24,26,31,42]. Finally, nine records reported specifically the animal species involved in their activities: in four cases two or three species were mentioned: goats, cattle and pigs [37], chickens and pigs [47], chickens and rabbits [21] or cattle, hens and alpacas [33]. In five records, authors referred only to one species: horses [27,35,39] or dairy cows [28,40].

### 3.5. Characteristics of the Users Involved

Only 40% of selected records specified that the users’ category involved psychiatric patients [25,30,51], people with autism spectrum disorders [33,49], elderly people with dementia [12,15,16,26], non-clinical population [27,31], drug users [23,35], depressed people [28,40], detainees [19] and long term unemployed [46]. In the remaining records (60%) the characteristics of the users involved were not described in detail. These records mainly provided a general definition such as “disabled” or “people with mental health problems” or rather they listed different categories of users for the same kind of intervention. Regarding users’ age, it is not possible to identify a real distribution because in the 48% of the records there is not a unique category of users or the age is not even considered. For the remaining records (52%), users are defined generically as adults (*n* = 12) [18,19,23,25,28,30,32,33,40,41,42,46], adolescents/young people (*n* = 4) [27,39,43,45], elderly (*n* = 4) [15,26,29,31] and children (*n* = 2) [34,48].

### 3.6. Kind of Human-Animal Interaction

**Figure 5:**
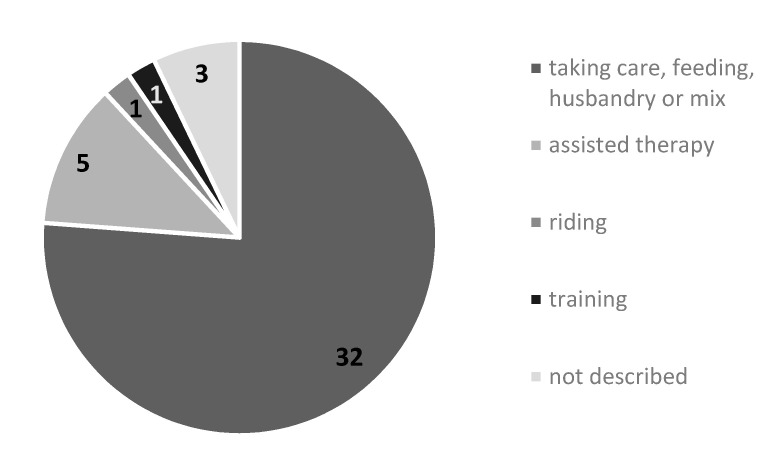
Categories of human–animal interactions (*n* = 42).

Seventy-six percent of records classified human–animal interactions as animal taking care, animal feeding, animal husbandry or a mix of these activities. In 12%, authors declared that users were involved in Animal Assisted Therapy [11,17,39,48,49]. Only one record referred to “riding horses” [27] and one to “training animals” [19] whereas in 7% human–animal interactions were not described in detail [13,22,50]. These results are summarized in Figure 5.

### 3.7. Other Activities without Animals

Eight records (19%) described green care programs based only on human–animal interaction [25,27,28,30,37,40,48,51]. In five records, authors declared some other activities but these were not described at all [19,20,32,45,46]. The 31% of records referred to mixed programs that included human–animal interactions together with agriculture, horticulture and gardening [10,13,17,18,21,22,23,24,33,36,38,42,44]. The remaining 37% described programs that combined human–animal interactions with extremely various activities such as playing, eating together and conventional therapies.

### 3.8. Animal Welfare and Health Perspectives

In 36 out of 42 records we analyzed, there is no reference to the health and welfare of the animals involved and the main focus was the benefit of the Green Care program for the human side. Only few authors described animal housing and husbandry conditions: herd and free access to pasture for horses [27,35,39], free-range stall for dairy cows [28]. Finally, only two records highlighted the need of a change of perspective, of improving the study of animal welfare to understand the impact of this kind of programs on animals [10,44]. Moreover, we noticed that no papers referred to the health risks for patients who interact with animals in a farm environment and there were no references to hygienic procedures and zoonosis control programs.

## 4. Discussion

Data analyzed in this review highlighted that the line between AAI and occupational therapy is often vague. The importance of the human–animal interaction is stressed in all the papers we included, but only 22 of them examined in detail how animals are beneficial in the Green Care context [25,26,27,28,29,30,31,32,33,34,35,36,37,38,39,40,41,42,43,44,45,46]. Six out of eight records focused only on the human–animal interaction in the Green Care context were published in Norway, a country where the research topic of Green Care with animals was developed between 2007 and 2014 providing social services to the community [25,27,28,30,40,51]. Certainly, the co-presence of the natural environment and interaction with animals is the main source of benefits for the patients/users, but none of the studies investigated the possible inner mechanisms involved. The selected timespan for the literature search results to be correct, as we did not find scientific literature dated before 2006. A book was published in that year whose five chapters are included in this review [9], highlighting that human–animal interactions in farm context were already carried out in practice, but the scientific interest for the topic developed later and only in some western countries with well-developed agro-economic systems.

Taking into account that papers were selected for reporting programs of Green Care with animals, as a matter of fact not every record discussed the benefits of these practices. In the majority of them, benefits were presented more as an opinion of the authors or the providers than as a scientific evidence [34,35,36,37,38,39,40,41,42,43,44,45,46,47,48,49,50,51]. Only six studies had an experimental design with a control group and a well-described methodology, which confirmed the efficacy of the Green Care approach with animals [25,26,27,28,29,30]. Generally, the main benefit described was an improvement of users’ well-being due to the non-institutional context. Anyway, the lack of internal and external validity of all papers analyzed addressed to the need of a more structured and well-designed research to investigate the real positive effects of Green Care.

We noticed that the descriptions of Green care programs’ settings were very generic. The word “farm” was used referring to a rural and productive context where many activities were provided to the beneficiaries. The only exception are “prison farms” which are well described by Furst [19]. The author investigated programs involving animals offered in the U.S. prisons: one of them was provided in a farm environment where inmates worked with farm animals. In some other records the word “farm” was replaced by “natural environment” without any additional description of the context. We can suppose that such kind of settings are not related to agriculture or livestock production and at the same time far away from the traditional idea of a “healthcare setting”.

Additionally, the animal species involved was not specified in the majority of records we analyzed. It seems that animal features are not so relevant in the Green Care context. Except for few studies focused on horses [27,35,39] and dairy cows [28,40], other animal species were not involved for their ethological characteristics but only for the rural context in which they were housed and their “non-human” nature. Without a priori choice of animal species, projects can be easily adapted to the users’ needs and will maintain flexibility for many beneficiaries’ categories.

Actually, users involved belonged to a wide range of categories including specific physical and mental disabilities [12,15,16,25,26,28,30,33,40,49,51], people with poor social inclusion [19,46], abused or affected by addiction [23,35]. More than a half of the records analyzed described human–animal interaction programs without specifying category of users, highlighting their great flexibility. Others, involving horses [27,35] and dairy cows [28,40], were focused on patients/users with specific features, fitting the animal species and the nature of the interaction with the standard therapeutic program.

In many cases, the kind of services provided with animals were not described in detail but generally referred to “animal taking care activities” (e.g., brushing, cleaning stables, feeding animals). These activities give new occupational skills to the users and increase their responsibility toward another living being, but they are far away from AAI, which are based on the development of a human–animal relationship [5], that is used as a tool to achieve therapeutic, educational or recreational goals, specifically established for each AAI project. In Green Care context, the non-judging interaction with animals is mostly limited to the possibility of physical contact, with an underspending of the AAI potentiality.

We highlighted that in 13 records human–animal interaction was not the only activity provided, but in the same context users could benefit from horticulture and gardening, which are relevant in Green Care [10,13,17,18,21,22,23,24,33,36,38,42,44]. This framework created an interconnection between “taking care of plants” and “taking care of animals” offering a holistic approach to the beneficiary who is completely surrounded by the rural environment.

Anyway, in the records we analyzed, we noticed lack of attention towards key issues such as animal welfare and health risks related to human–animal interactions in rural context. Interactions with beneficiaries with disabilities and without specific knowledge and skills on animal husbandry could be a source of stress for animals, especially when not well managed and trained by animal handlers [52]. If precautions are not taken, the interaction with humans can be physically and mentally demanding for animals [53]. Poor description of animal housing and husbandry, as well as the absence of clear reference to animal welfare status and monitoring, suggest the need to raise awareness among practitioners towards a more ethical approach in the involvement of animals in such kind of activities. Moreover, health issues related to zoonosis or pathogens with antimicrobial resistance transmissible to humans in rural contexts is another crucial topic due to the possible vulnerability of users’ health. Hygienic procedures and health monitoring of farm animals were not mentioned at all in the records included in this review, highlighting an underestimation of the problem.

Finally, given the heterogeneity of information sources and the type of studies included, the methodology and level of detail provided by each record are various; this factor can represent a limitation of this review. However, the question underlying this review was not focused on the effectiveness of GC with animals, but more on the description of this emerging phenomenon. Therefore, it was considered to include also different types of information sources: as previously reported, of the 42 included voices, 33 are peer reviewed journal articles, six are book chapters published by Springer, one is a peer reviewed conference proceeding, one is a book, and one is a report. For all these records, it was possible to identify the study purposes (as described in 3.2), which in some cases have been achieved through qualitative methods, in other cases through quantitative ones. In particular, as given ahead, only six of them had an experimental design with a control group and a well-described methodology [25,26,27,28,29,30]. Nevertheless, by adopting a critical perspective on the selected literature, some considerations may arise. Analyzing the characteristics of the records included, the studies often lack relevant information: on one hand, the detailed description of patients (type of disease, age, etc.), operators and animals involved (species, type of training and management, etc.); on the other hand, the specification of the activities that are carried out during the interaction with animals. In the interests of increased consistency of the scientific literature which investigates the phenomenon, in particular as regards efficacy studies, the purpose of standardizing the design and reporting of the studies conducted can undoubtedly contribute to shed light on the complex interaction between humans, animals and the environment in a one health/one welfare perspective.

## 5. Conclusions

From our literature review we can conclude that interaction with animals in the Green Care Context is present and extremely various. The countries of publication of the records included demonstrate that these practices are typical of developed areas. In some studies, the human–animal interaction is minimal, but in others is connected properly to a therapeutic goal and could be considered as an AAI (even if we do not know if in these cases there was an adequate support of professionals). Moreover, we have to say that when studies describe the benefits deriving from Green Care with animals it is difficult to distinguish which of these come from the human–animal interaction or from the natural environment. The incorporation of AAI into Green Care will benefit form a greater awareness of the animal species involved and its ethology; for future research, it is desirable that more attention will be paid to the planning of interventions, to animal welfare and the health aspects of the interaction, in order to guarantee safety for both users and animals.

## Figures and Tables

**Table 1:** Search strings in Web of Science and Google Scholar.

	Web of Science
613	**TI** = ((care farm* OR social farm* OR social agriculture OR farm assisted OR green care OR nature based rehabilitation OR nature assisted therap* OR working in nature OR wilderness therap* OR farm based OR farm therap* OR rural welfare) AND (farm animal* OR animal* OR assisted intervention* OR animal assisted OR horse* OR donkey* OR cow* OR goat* OR sheep OR rabbit* OR chicken* OR pig* OR camelid* OR human–animal)) OR **TS** = ((“care farm*” OR “social farm*” OR “social agriculture” OR “farm assisted” OR “green care” OR “nature based rehabilitation” OR “nature assisted therap*” OR “working in nature” OR “wilderness therap*” OR “farm based” OR “farm therap*” OR “rural welfare”) AND (“farm animal*” OR animal* OR “assisted intervention*” OR “animal assisted” OR horse* OR donkey* OR cow* OR goat* OR sheep OR rabbit* OR chicken* OR pig* OR camelid* OR “human–animal”))
	*Databases = WOS, KJD, MEDLINE, RSCI, SCIELO Timespan = 2000–2020; Search language = Auto* **TI** = title **TS** = topic
	**Google Scholar**
400	care farm OR social farm OR social agriculture OR farm assisted OR green care OR nature based rehabilitation OR nature assisted therapy OR working in nature OR wilderness therapy OR farm based OR farm therapy AND farm animal OR animal OR assisted intervention OR animal assisted OR human– animal *Timespan = 2000–2020; Search language = Auto*

## Data Availability

Not applicable.

## Appendix A

**Table A1:** List of literature included in chronological order.

**Author**	**Title**	**Category**	**Journal/Book Name**	**Year**	**Country**
Relf, Paula Diane	Agriculture and Health care—The care of plants and animals for therapy and rehabilitation in the United States	Book Chapter	Farming for Health	2006	United States of America
Wiesinger, Georg; Neuhauser, Fritz and Putz, Maria	Farming for Health in Austria—Farms, horticultural therapy, animal-assisted therapy	Book Chapter	Farming for Health	2006	Austria
Neuberger, Konrad; Stephan, Ingrid; Hermanowski, Robert; Flake, Albrecht; Post, Franz-Joseph and Van Elsen, Thomas	Farming for Health: Aspects from Germany	Book Chapter	Farming for Health	2006	Germany
Abramsson, Karin and Tenngart, Carina	Nature and Health in Sweden	Book Chapter	Farming for Health	2006	Sweden
Furst, Gennifer	Prison-Based Animal Programs a National Survey	Journal Article	The Prison Journal	2006	United States of America
van Elsen, Thomas; Gunther, Amelie and Pedroli, Bas	The contribution of care farms to landscapes of the future—A challenge of multifunctional agriculture	Book Chapter	Farming for Health	2006	Germany
Hassink, Jan; Zwartbol, Ch.; Agricola, H. J.; Elings, M. and Thissen J. T. N. M.	Current status and potential of care farms in the Netherlands	Journal Article	NJAS—Wageningen Journal of Life Sciences	2007	The Netherlands
Chalfont, Garuth	Design for nature in dementia care	Book	Design for Nature in Dementia Care	2007	United Kingdom
Berget, Bente; Skarsaune, Ingvild; Ekeberg, Oivind and O. Braastad, Bjarne	Humans with Mental Disorders Working with Farm Animals	Journal Article	Occupational Therapy in Mental Health	2007	Norway
Berget, Bente; Ekeberg, Oivind and O. Braastad, Bjarne	Animal-assisted therapy with farm animals for persons with psychiatric disorders: effects on self-efficacy, coping ability and quality of life, a randomized controlled trial	Journal Article	Clinical Practice and Epidemiology Mental Health	2008	Norway
Berget, Bente; Ekeberg, Oivind and O. Braastad, Bjarne	Attitudes to animal-assisted therapy with farm animals among health staff and farmers	Journal Article	Journal of Psychiatric and Mental Health Nursing	2008	Norway
Hine, Rachel; Peacock, Jo and Pretty, Jules	Care farming in the UK: Evidence and Opportunities	Report	National Care Farming Initiative	2008	United Kingdom
De Bruin, Simone R.; Oosting, Simon J.; Kuin, Yolande; Hoefnagels, Erica C. M.; Blauw, Ypie H.; De Groot, Lisette C. P. G. M. and Schols, Jos M. G. A.	Green Care Farms Promote Activity Among Elderly People With Dementia	Journal Article	Journal of Housing For the Elderly	2009	The Netherlands
de Bruin, Simone; Oosting, Simon J.; Tobi, Hilde; Blauw, Y. H.; Schols, Jos M. G. A. and De Groot, C. P. G. M.	Day care at green care farms: A novel way to stimulate dietary intake of community-dwelling older people with dementia?	Journal Article	The Journal of Nutrition Health and Aging	2010	The Netherlands
Ferwerda-van Zonneveld, R. T.; Oosting, S. J. Additionally, Kijlstra, A.	Care farms as a short-break service for children with Autism Spectrum Disorders	Journal Article	NJAS—Wageningen Journal of Life Sciences	2012	The Netherlands
Pedersen, Ingeborg; Martinsen, Egil Wilhelm; Berget, Bente and O. Braastad, Bjarne	Farm Animal-Assisted Intervention for People with Clinical Depression: A Randomized Controlled Trial	Journal Article	Anthrozoös	2012	Norway
Pedersen, Ingeborg; Ihlebæk, Camilla and Kirkevold, Marit	Important elements in farm animal-assisted interventions for persons with clinical depression: a qualitative interview study	Journal Article	Disability and Rehabilitation	2012	Norway
Burgon, Hannah Louise	Horses, mindfulness and the natural environment: Observations from a qualitative study with at-risk young people participating in therapeutic horsemanship	Journal Article	The International Journal of Psychosocial Rehabilitation	2013	United Kingdom
Leck, Chris; Evans, Nick and Upton Dominic	Agriculture—Who cares? An investigation of ‘care farming’ in the UK	Journal Article	Journal of Rural Studies	2014	United Kingdom
Chawla, Louise	Children’s Engagement with the Natural World as a Ground for Healing	Book Chapter	Greening in the Red Zone: Disaster, Resilience and Community Greening	2014	United States of America
Hauge, Hilde; Kvalem, Ingela L.; Berget, Bente; Enders-Slegers, Marie-José and O. Braastad Bjarne	Equine-assisted activities and the impact on perceived social support, self-esteem and self-efficacy among adolescents—an intervention study	Journal Article	International Journal of Adolescence and Youth	2014	Norway
Granerud, Arild and Eriksson, Bengt G.	Mental Health Problems, Recovery, and the Impact of Green Care Services: A Qualitative, Participant-Focused Approach	Journal Article	Occupational Therapy in Mental Health	2014	Norway
Iancu, Sorana C.; Zweekhorst, Marjolein B. M.; Veltman, Dick J., van Balkom, Anton J. L. M. and Bunders, Joske F. G.	Mental health recovery on care farms and day centres: A qualitative comparative study of users’ perspectives	Journal Article	Disability and Rehabilitation	2014	The Netherlands
Kogstad, Ragnfrid Eline; Agdal, Rita and Hopfenbeck, Mark Steven	Narratives of Natural Recovery: Youth Experience of Social Inclusion through Green Care	Journal Article	International Journal of Environmental Research and Public Health	2014	Norway
Loue, Sana; Karges, Richard R. and Carlton, Candace	The Therapeutic Farm Community: An Innovative Intervention for Mental Illness	Journal Article	Procedia—Social and Behavioural Sciences	2014	Romania
Leck, Chris; Upton Dominic and Evans, Nick	Growing well-beings: The positive experience of care farms	Journal Article	British Journal of Health Psychology	2015	United Kingdom
Kučera, Zdeněk	Social Agriculture—Alternative Type of Production	Proceedings	The International Scientific Conference INPROFORUM 2015	2015	Czech Republic
Ellingsen-Dalskau, Lina Harvold; Morken, Margrete; Berget, Bente and Pedersen, Ingeborg	Autonomy support and need satisfaction in prevocational programs on care farms: The self-determination theory perspective	Journal Article	Work	2016	Norway
Pedersen, Ingeborg; Dalskaua, Lina Harvold; Ihlebæk, Camilla and Patil, Grete	Content and key components of vocational rehabilitation on care farms for unemployed people with mental health problems: A case study report	Journal Article	Work	2016	Norway
Ellingsen-Dalskau, Lina Harvold; Berget, Bente; Pedersen, Ingeborg; Tellnes, Gunnar and Ihlebæk, Camilla	Understanding how prevocational training on care farms can lead to functioning, motivation and well-being	Journal Article	Disability and Rehabilitation	2016	Norway
Hemingway, Ann; Ellis-Hill, Caroline and Norton, Elizabeth	What does care farming provide for clients? The views of care farm staff	Journal Article	NJAS—Wageningen Journal of Life Sciences	2016	United Kingdom
Hassink, Jan; de Bruin, Simone R.; Berget, Bente and Elings Marjolein	Exploring the Role of Farm Animals in Providing Care at Care Farms	Journal Article	Animals	2017	The Netherlands/Norway
Anderson, Keith A.; Chapin, Kate P.; Reimer, Zachary and Siffri, Gina	On fertile ground: An initial evaluation of green care farms in the United States	Journal Article	Home Health Care Services Quarterly	2017	United States of America
Jeong, Sun Jin; Hassink, Jan; Gim, Gyung Mee; Park, Sin-Ae and Kim, Seon Ok	Status and Actual Condition Analysis for Current Operational Cases of Care Farms in South Korea	Journal Article	Journal of People, Plants, and Environment	2017	South Korea
Gorman, Richard	Therapeutic landscapes and non-human animals: the roles and contested positions of animals within care farming assemblages	Journal Article	Social & Cultural Geography	2017	United Kingdom
Therkildsen Sudmann, Tobba	Communitas and Friluftsliv: equine-facilitated activities for drug users	Journal Article	Community Development Journal	2018	Norway
Ibsen, Tanja Louise; Eriksen, Siren and Grindal Patil, Grete	Farm-based day care in Norway—a complementary service for people with dementia	Journal Article	Journal of Multidisciplinary Healthcare	2018	Norway
Lee, A-Young; Oh, Yun Ah; Kim, Seon Ok; Kim, Dae-Sik and Park, Sin-Ae	Survey on Demand and Operation Status of Care Farms in South Korea	Journal Article	Journal of People, Plants, and Environment	2018	South Korea
Gagliardi, Cristina; Santini, Sara; Piccinini, Flavia; Fabbietti, Paolo and di Rosa, Mirko	A pilot programme evaluation of social farming horticultural and occupational activities for older people in Italy	Journal Article	Health and Social Care in the Community	2019	Italy
Hassink, Jan; Vaandrager, Lenneke; Buist, Yvette and de Bruin, Simone	Characteristics and Challenges for the Development of Nature-Based Adult Day Services in Urban Areas for People with Dementia and Their Family Caregivers	Journal Article	International Journal of Environmental Research and Public Health	2019	The Netherlands
Torquati, Biancamaria; Stefani, Gianluca; Massini, Giulio; Cecchini, Lucio and Chiorri Massimo	Social farming and work inclusion initiatives for adults with autism spectrum disorders: A pilot study	Journal Article	NJAS—Wageningen Journal of Life Sciences	2019	Italy
Gorman, Richard	Thinking critically about health and human–animal relations: Therapeutic affect within spaces of care farming	Journal Article	Social Science & Medicine	2019	United Kingdom
